# The associated expression of Maspin and Bax proteins as a potential prognostic factor in intrahepatic cholangiocarcinoma

**DOI:** 10.1186/1471-2407-6-255

**Published:** 2006-10-26

**Authors:** Antonello A Romani, Paolo Soliani, Silvia Desenzani, Angelo F Borghetti, Pellegrino Crafa

**Affiliations:** 1 Dipartimento di Medicina Sperimentale – Sezione di Patologia Molecolare ed Immunologia, Università degli Studi di Parma, 43100 Parma, Italy; 2 Dipartimento di Scienze Chirurgiche – Sezione di Clinica Chirurgica Generale e dei Trapianti d'Organo, Università degli Studi di Parma, 43100 Parma, Italy; 3 Dipartimento di Patologia e Medicina di Laboratorio – Sezione di Anatomia ed Istologia Patologica, Università degli Studi di Parma, 43100 Parma, Italy

## Abstract

**Background:**

Maspin, a member of the serpin family, is a suppressor of tumor growth, an inhibitor of angiogenesis and an inducer of apoptosis. Maspin induces apoptosis by increasing Bax, a member of the Bcl-2 family of apoptosis-regulating proteins. In this exploratory study, we investigated the associated expression of Maspin and Bax proteins as a potential prognostic factor in intrahepatic cholangiocarcinoma (IHCCA).

**Methods:**

Twenty-two paraffin-embedded samples were analyzed by immunohistochemical methods using Maspin, Bax and CD34 antibodies. Maspin was scored semiquantitatively (HSCORE). Apoptosis was assessed using an antibody against cleaved caspase-3.

**Results:**

The strong relationship observed between the expression of Maspin and Bax, indicates that Bax is likely to be the key effector of Maspin-mediated induction of apoptosis as indicated by the activation of cleaved caspase-3. We categorized Maspin HSCORE by calculating the optimal cutpoint. A Maspin HSCORE above the cutpoint was inversely related with tumor dimension, depth of tumor and vascular invasion. Uni/multivariate analysis suggests that a Maspin HSCORE below the cutpoint significantly worsens the patients' prognosis. Tumors with Maspin HSCORE below the cutpoint had a shorter survival (11+/-5 months) than did patients with Maspin HSCORE above the cutpoint (27+/-4 months), whereas Kaplan-Meier analysis and logrank test showed no significant difference in overall survival between the patients.

**Conclusion:**

The associated expression of Maspin and Bax might delay tumor progression in IHCCA. Maspin above the cutpoint might counteract tumor development by increasing cell apoptosis, and by decreasing tumor mass and cell invasion. The combined expression of Maspin and Bax appears to influence the susceptibility of tumor cholangiocytes to apoptosis and thus may be involved in delaying IHCCA progression.

## Background

Cholangiocarcinoma is a rare tumor that is difficult to diagnose and is associated with a high mortality [1-3]. The term cholangiocarcinoma referred originally to just primary tumors of the intrahepatic bile ducts, while now it is regarded as inclusive of intrahepatic, perihilar, and distal extrahepatic tumours of the bile ducts [4]. The incidence of intrahepatic bile duct carcinoma (IHCCA) is increasing worldwide, although the cause for this rise is unclear [1,2]. It could be related to an interplay between predisposing genetic factors and environmental triggers. Surgical resection is the only curative option for cholangiocarcinoma, particularly for extrahepatic ones, with results depending on technique and patient selection [5-9]. Chemotherapy and radiotherapy are ineffective in patients with inoperable tumors. and biliary drainage is the mainstay of palliation [10,11]. Over the last ten years, considerable effort has been devoted to the search for markers to improve the diagnosis and the management of CCA. One of the authors (AAR) has developed a Perl [12] script for differential *in silico *screening between cancer and normal EST tissue libraries devoted to the identification of differentially expressed genes. **E**xpressed **S**equence **T**ag (**EST**) sequencing [13] is of value in discovering new genes (or rather markers), and provides an inexpensive approach to identifying large numbers of new genes [14,15]. These can be used in gene differential expression screening procedures. The widely recognized contribution of ESTs to gene discovery has spurred the production of very large numbers of these sequences. There are currently over 6.0 × 10^6 ^human EST sequences in the public databases [16] and ESTs represent more than 60% of the Gene Bank database entries. Using this procedure, a pool of mRNA sequences were identified that, on a statistical basis [17,18], are preferentially expressed in hepatocarcinoma and cholangiocarcinoma (data not shown). We focused our attention on those over-expressed genes known to counteract or delay tumor progression. Among these genes, we selected the Maspin gene whose protein product belongs to the family of protease inhibitors, and has been described as an apoptosis-sensitizing protein [19].

Maspin (**ma***mmary ***s***erine ***p***rotease ***in***hibitor*) is a member of the serpin (***ser****ine ****p****rotease ****in****hibitor*) family [20,21] and has recently been described as a clinically relevant factor in several types of tumor [22-25]. A decreased expression of Maspin has been related to the progression of breast cancer, and the loss of its expression is regarded as a critical step during the transition from non-invasive to invasive breast cancer [26,27]. It has been suggested that Maspin could be a reliable predictor for metastatic disease [28] and a clinical relevant inhibitor of tumor progression [29].

The mechanism of Maspin action is still controversial and it could act as an inhibitor of angiogenesis[30,31], and suppressor of tumor growth and metastasis *in vivo *[32,33]. There is increasing evidence that in addition to its anti-angiogenic effect, Maspin can induce apoptosis of tumor cells. Moreover, Maspin over-expression modulates tumor cell apoptosis through the regulation of Bcl-2 family proteins [34] resulting in an increased release of cytochrome C from mitochondria [35] and an increased apoptosis in Maspin-expressing cells [36]. Other work showed that in Maspin- expressing cells, the Bcl-2 level was decreased and the level of pro-apoptotic protein Bax was significantly increased In a recent study [19], the authors obtained cellular, molecular, and biochemical evidence that supports a key role for Bax in sensitization of Maspin-mediated apoptosis. Thus, Bax over-expression may predict an elevated cellular apoptotic response to a broad spectrum of apoptotic stimuli.

To our knowledge, the combined role of Maspin and Bax in IHCCA has been not investigated. The aim of this exploratory study was to assess the associated expression patterns of Maspin and Bax in specimens of IHCCA and to investigate whether Maspin and Bax might be used as novel prognostic factors.

## Methods

### Patient selection

From January 1997 to December 2005, 33 patients affected by cholangiocarcinoma were admitted to the Surgery department. Five patients did not undergo surgery because of advanced disease, advanced liver cirrhosis or poor general condition. The remaining 28 patients (9 males and 19 females) with an average age of 68 years (range: 36–79), underwent liver resection for cholangiocarcinoma at the Dipartimento di Scienze Chirurgiche, Sezione di Clinica Chirurgica Generale e dei Trapianti d'Organo. Of these 28 patients, 22 had IHCCA and six patients had hilar extrahepatic cholangiocarcinoma (Klatskin): 3 patients of type IIIb, 2 had a peripheral type and one had a periductal type according to the Bismuth-Corlette indication.

We focused our attention on IHCCA, since the epidemiological data, retrieved from the Parma Tumor Registry (Divisione di Oncologia Medica, Azienda Ospedaliera di Parma), showed a marked increase in IHCCA in our province from 1–2 cases/year in 1994 to 9–12 cases/year in 2002–2004.

Background information including clinico-pathologic data, and patients' survival was retrieved from histologic and clinical records. None of the patients had received chemotherapy or radiotherapy prior to surgery. All surgical procedures were performed with radical intent (R0 after surgery). Liver needle biopsies of five patients included in this study were available.

The study was conducted in accordance with the tenets of the Declaration of Helsinki. Following the indication of Italian DLgs no. 196/03 (Codex on Privacy), every precaution has been taken to respect the privacy of the patients and the confidentiality of the patient's information. Written consent to use stored tissue was obtained from all the living patients.

### Sample sources

The samples were retrieved from the archived files of the Dipartimento di Patologia e Medicina di Laboratorio – Sezione di Anatomia ed Istologia Patologica. Formalin- fixed, paraffin-embedded, hematoxylin-eosin-stained sections were reviewed and the pathologist (PC) re-staged the samples according to the recent AJCC classification [37]. Sections with poorly differentiated tumor areas with adjacent normal and/or dysplastic tissues were then selected for immunohistochemical studies.

### Immunohistochemistry

The paraffin sections were dewaxed, rehydrated, washed in Tris-buffered saline (TBS) (150 mmol/L NaCl, 50 mmol/L Tris, pH 7.4). The antigen determinants masked by formalin-fixation and paraffin-embedding were exposed by heating the section in a microwave oven for 10 min in an *epitope retrieval solution *(10 mM Tris Base, 1 mM EDTA solution, 0.05% (v/v) Tween 20, pH 9.0). The sections used for the pan-endothelial-cell marker CD34 were treated with 1 mg/ml protease type XXIV [38] (Sigma-Aldrich, Inc., St. Louis, MO). The endogenous peroxidase activity was quenched with 3% H_2_O_2 _in deionized water for 10 min. Non-specific bindings were blocked in TBS containing 5% acetylated albumin (Aurion, Wegeningen, The Netherlands), 5% normal mouse serum (Dako Corp., Carpinteria, CA) and 0.1% porcine gelatin type B (Sigma-Aldrich). The primary antibodies against Maspin (Novocastra, Newcastle upon Tyne, UK), Bax and CD34 (Santa Cruz Biotechnology, Inc. Santa Cruz, CA), and cleaved caspase-3 (Cell Signaling Technology Inc., Beverly, MA) were used at 1:35, 1:75, 1:50 and 1:200 dilutions, respectively. Immunoperoxidase staining was performed using an anti-Mouse/Rabbit Poly HRP Detection Kit (Chemicon International, Temecula, CA) according to the manufacture's specifications and developed with DAB. The specimens were counterstained with hematoxylin, mounted and examined by light microscopy. Routine negative controls used TBS instead of the primary antibody. An isotype control was conducted using a mouse IgG isotype control serum (Dako Corp., Carpinteria, CA). Negative and isotype controls were used in all staining runs. All negative and isotype controls resulted in negative immunohistochemical reactions. Adjacent normal and/or dysplastic areas were selected as internal controls. As positive controls, sections from tumors known to express Maspin and/or Bax were used (according to the manufacture's datasheet). A preparation of excised inflammatory tonsils was used to asses cleaved caspase-3 expression.

The staining intensities of the IHCCAs and liver fine-needle biopsies were compared with those of adjacent non-neoplastic biliary ducts and by semiquantitative visual evaluation by three observers (PC, AAR and SD). Overexpression was considered to be present when the intensity of tumor staining was at least twice the staining intensity of adjacent non-neoplastic areas.

### Image analysis

The expression of Maspin was evaluated using a microscope image analysis system (Nikon Digital Net system). For each patient, five randomized, non-overlapping rames were evaluated in the same neoplastic area at 400× field magnification (0.198 mm^2^·field). At least 50 cells were counted for a total of 250–500 cells analyzed. Toassess reproducibility of the image analysis, the pathologist (PC) blindly scored the 66% of the total of patients and also randomly selected frames.

### Quantification of expression

Maspin and Bax immunopositivities were semi-quantitatively evaluated. This method described by McCarty [39] is referred to as *HSCORE*. The percentage of positive cells for Maspin or Bax, respectively, was linked with the intensity of staining classified into four groups: ***0***-negative; ***1***-slight positive (light brown color of the either homogeneously or spotty stained cells); ***2***-distinct positivity (medium brown homogeneously stained cells); ***3***-strong positivity (dark brown to black homogeneously stained cells).

We calculated the HSCORE using this formula:

**HSCORE **= ∑P_i_·(j+1),

where P_i _is the percentage of positive cells in the sample investigated, and j is one of the four positivity grades (0–3) defined above.

### Quantification of neoplastic cells expressing cleaved caspase-3

Cleaved caspase-3 positive neoplastic cells were quantified using a microscopic grid at 400× magnification and expressed as percentages of all tumor cells present. Moreover, we carefully separated cleaved caspase-3 positive lymphocytes and intratumoral macrophages from phagocytosed apoptotic debris. Apoptotic cells from highly necrotic areas were excluded from the analysis.

### Evaluation of microvessel density

The procedure used was in accordance to the international consensus method of intratumoral microvessel density assessment [40] by using the pan-endothelial CD34 marker. The assessment was conducted according to our recently published procedure [41]. For microvessel quantitation, the tumoral areas containing the highest number of capillaries and small vessels were identified by light microscopy at low power magnification. In each tumor, three areas with the highest vascularization were identified, and a microvessel count was performed at x200 magnification. Although agreement was not exact in every case, no significant variation between the investigators' counts (PC, AAR, SD) was noted.

### Statistical analysis

The biomarker and clinical data were analyzed using S-Plus Professional Edition v.6.1 (Insightful Corp., Seattle, WA). Fisher's Exact Test was used to test the hypothesis of independence between categorical variables in 2 × 2 tables. The ANOVA procedure was used to evaluate the association between ordinal and categorical variables. All tests were two-sided. The significance values higher than 0.05 and less than 0.10 indicated a trend. The conversion of a continuous covariate into a binary one when there is no established cut-off point (previous published results or biological knowledge) was performed by using an outcome-oriented statistical method (such as the optimal cutpoint estimation).

For optimal cutpoint estimation a corrected P value method, as described by Hothorn and Lausen [42], was used. Estimates of the survival probability were calculated using the Kaplan-Meier method, and the logrank test was employed to test the null hypothesis of equality in overall survival among patients with Maspin above or below the cutpoint. The hazard ratio associated with Maspin expression above or below the cutpoint was estimated by logistic regression.

## Results

### Demographic data

This exploratory study concerned a relatively short period of inclusion (7 years) for this tumor, with a satisfactory homogeneity of patients treated with radical intent. T3he average age of the 15 females and 7 males undergoing surgery for IHCCA was 68 years (range 52–79). Two patients out of 22 were positive to C viral hepatitis (one of them showed B and C infection). None of the other patients had chronic liver disease. At presentation, twelve patients had one comorbidity (diabetes, hypertension or heart disease), eight patients had two comorbidities (mainly diabetes and hypertension), and only two patients had three comorbidities (chronic obstructive lung disease, diabetes and heart disease).

The surgical procedures, performed by one of the authors (PS), for IHCCA included: 5 right trisectionectomies, 4 right and 3 left hemihepatectomies (including the extended), 7 sectoriectomies (anterior, posterior and lateral) and 3 segmentectomies. The mean liver volume removed was 49 ± 19.2% (range 22–75%) while 4.5 ± 1.5 were the mean number of resected hepatic segments.

Seventeen (77%) out of 22 patients with IHCCA were characterized as having well to moderate differentiation, and 5 (23%) poor differentiation. The tumor growth pattern was classified as papillary in five cases, glandular in six, and solid in nine. Patient characteristics are summarized in Table 1 according to Maspin HSCORE cutpoint.

### Immunohistochemical detection of Maspin, Bax and cleaved Caspase-3

The immunohistochemical staining, performed with anti-Maspin, anti-Bax monoclonal antibody and anti-cleaved-caspase 3 polyclonal antibody respectively, produced readily quantifiable results.

**Figure 1:**
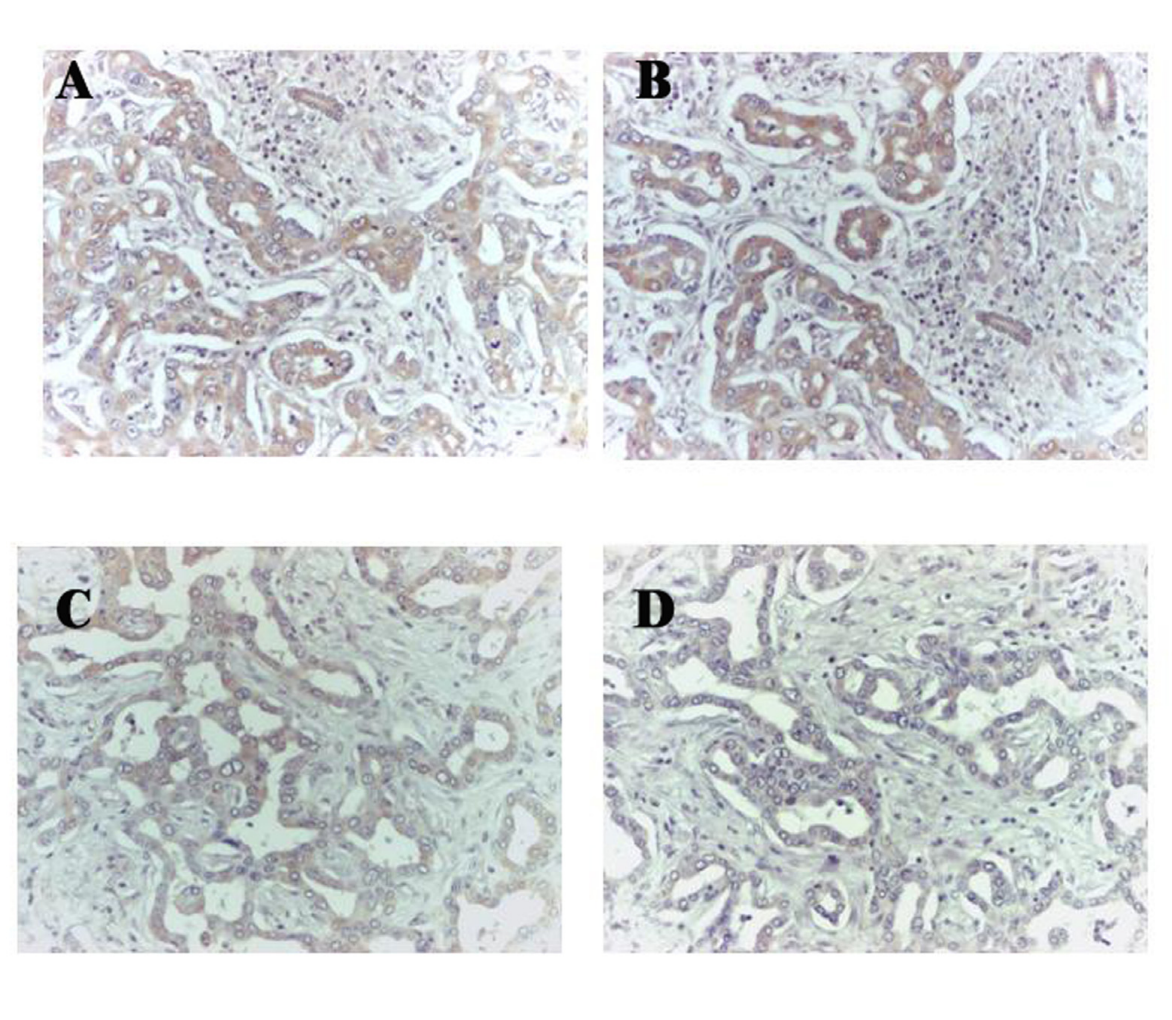
**Immunoperoxidase staining of serial paraffin-embedded tissue sections of IHCCA of Maspin and Bax shows their expression in cholangiocarcinoma cells (brown staining)**. Panels A and B refer to a 79-year-old female, Maspin HSCORE = 111 and Bax HSCORE = 95; panels C and D refer to a 71-year-old male, Maspin HSCORE = 70 and Bax HSCORE = 47.

None of the samples exhibited Maspin expression in normal biliary tract epithelium (internal negative control). In comparison, Maspin expression was present in 77% (17/22) of IHCCA examined (see Figure 1A,C) and absent in five patients (23%) In the sections analyzed, the number of positive cells ranged from 0% to 61%, while the intensity varied from negative specimens (grade 0) to dark brown (grade 3). The Maspin-immunopositive cells basically showed three morphological staining patterns: (a) cytoplasmatic (spotted or homogeneous), (b) nuclear, and (c) both. The immunoreactivity for Maspin was completely negative in normal hepatocytes and in normal/displastic biliary duct far from IHCCA border and in all hepatocarcinoma tested (data non shown). We note, however, that in the analyzed IHCCA, the expression of Maspin was present in those hepatocytes and/or displastic biliary duct cells that surround the neoplastic areas. Moreover, in the Maspin-positive areas examined, the presence of immunopositivity in the bile duct secretion, and a reinforcement of positivity along the luminal surface of tumor bile ducts were sometimes observed.

The Maspin immunopositivities were semi-quantitatively evaluated as described in the Methods section. Maspin HSCORE ranged from 0 to 181.1 with a mean value of 71.3 ± 63.7 (median 69.7). The standardized skewness and kurtosis values (0.723 and -1.426, respectively) are within the range expected for data from a normal distribution.

Maspin is regarded as an apoptosis-sensitizing protein [19]. The link between Maspin expression and apoptosis induction has been explained by the regulation of pro- apoptotic Bcl-2 family proteins [34], resulting in predominantly increased Bax expression.

Bax immunostaining was cytoplasmic and homogeneous. We observed clear-cut expression of Bax in 45.5% (10/22) of the evaluated specimens. In positive samples, it was expressed in more than 75% of the neoplastic cells (see Figure 1B,D). Interestingly, when Bax was expressed we always observed Maspin expression (*P- value *= 0.039). Bax HSCORE ranged from 0 to 248.1 with a mean value of 67.9 ± 96.5. Only the kurtosis value (0.036) was within the range expected for data from a normal distribution.

**Figure 2:**
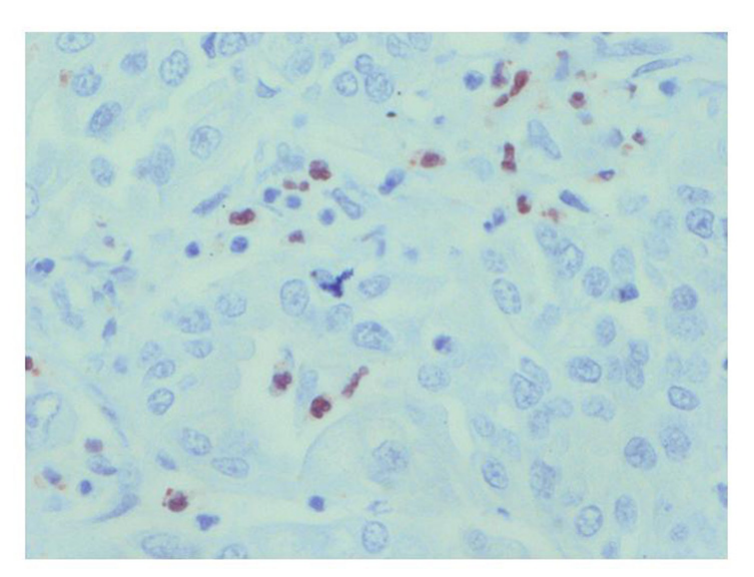
**Immunoperoxidase staining of apoptosis (cleaved caspase-3) in IHCCA tissue sections (brown staining)**. Immunohistochemical detection of cleaved caspase-3 in IHCCA specimens: Active caspase-3 staining displays a cytoplasmatic and/or nuclear pattern in neoplastic cells without morphological features of apoptosis.

The associated expression of Bax in Maspin positive sections might imply an activation of the apoptotic pathway. Considering the central role of caspase-3 activation in apoptosis, we then examined the occurrence of cleaved caspase-3 in our Bax-positive IHCCA tissue samples. In 6 out 10 cases, cleaved caspase-3-positive neoplastic cells were observed, albeit in a very limited number of cells. The percentage of active caspase-3-positive tumor cells ranged from 1 to 5%, displaying cytoplasmatic and/or nuclear staining pattern (see Figure 2). The cytoplasmatic staining showed two different patterns: spotted, granular or homogeneous, while the intensity varied from light to dark brown. The appearance of the active form of caspase-3 in the cytoplasm of the neoplastic cells undergoing apoptosis is an early event that precedes the development of the classical morphological features of apoptosis.

In Bax-positive samples, cleaved caspase-3-positive infiltrating lymphocytes were detected, serving as an internal positive control. In some cases, granular cytoplasmic staining in macrophages was also observed, probably representing phagocytosed apoptotic debris.

### Statistical correlation between Maspin HSCORE and clinical characteristics

**Figure 3:**
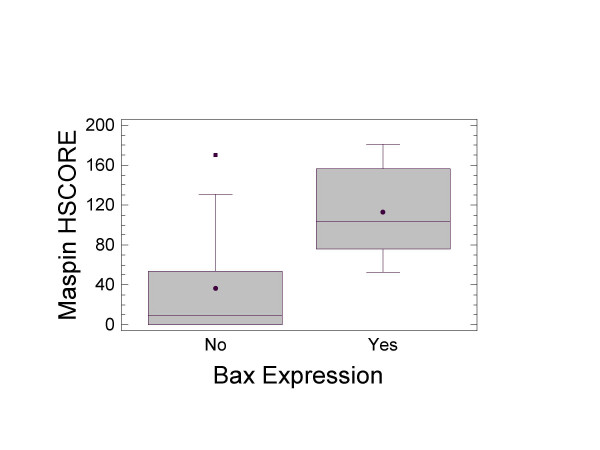
***Box-and-whisker *plot for the presence or absence of Bax expression versus Maspin HSCORE**. The solid black circles represent the mean values. The solid black represent outlier patients with Maspin HSCORE outside the 95% CI.

**Figure 4:**
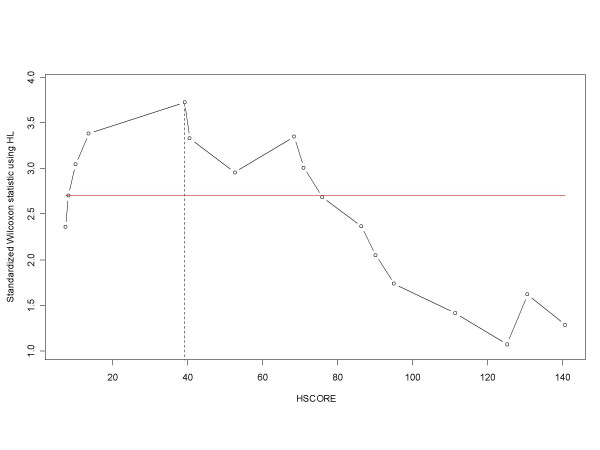
**Optimal cutpoint determined using an approximation for small samples**. Optimal cutpoint determined using an approximation for small samples as described. The highest value, discriminant with respect to the occurrence of Bax expression, is 39.12

Due to the small size of the cohort examined our study should be viewed as exploratory. However, in the following correlative analyses, the *P *values are reported as an indication of confidence in the results, and will be used to prioritize further testing. As shown in Figure 3, in the absence of Bax the mean Maspin HSCORE value was of 36.6 ± 14 compared with 113.1 ± 16 when Bax was present (*P-value *= 0.0035). It is noteworthy that there was a linear relationship between Maspin and Bax HSCOREs (*P-value *= 0.0425). We then decided to transform the continuous variable Maspin HSCORE into a categorical one (binary variable) to make the model more interpretable. By using the statistical procedure [42] described in the Materials and Methods section, the lowest value of the Maspin HSCORE that can discriminate the presence of Bax was 39.12 (Figure 4, *P*-value = 0.00075). The Maspin HSCORE was therefore categorized into two classes: above and below the cutpoint. The two groups of patients were comparable with respect to age, sex, grade, nodal involvement and number of hepatic segments resected.

**Figure 5:**
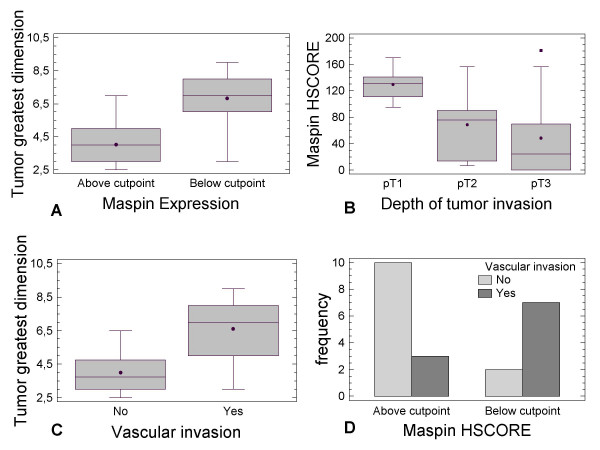
**Maspin HSCORE versus tumor parameters**. *Box-and-whisker *plots of Maspin HSCORE above/below cutpoint and tumor dimension (A), of Maspin HSCORE versus depth of tumor invasion (B), and Maspin HSCORE versus vascular invasion (C). The open squares represent those outlier patients outside the 95% confidence interval. The black solid circles represent the mean values, while the line inside the box represents the median. (D) Bar-chart plot of patients with Maspin HSCORE above/below cutpoint versus vascular invasion.

Maspin HSCORE was inversely correlated with gross neoplastic mass, estimated by the greatest tumor dimension (*P-value *= 0.0096) (see Figure 5A). The patients with Maspin expression above the cutpoint had a mean tumor mass of 4.26 ± 0.47 cm versus 6.83 ± 0.56 cm for those patients below the cutpoint. In particular, it is of note that the largest tumor mass was seen in the absence of Maspin and Bax (mean value = 6.9 ± 1.02 cm); the Maspin expression (without a concomitant Bax expression) was associated with a smaller tumor mass (mean value = 5.36 ± 2.66 cm), while the simultaneous presence of Maspin and Bax reduced further the mean tumor mass to 4.1 ± 1.4 cm. Student's *t*-test revealed that only the simultaneous expression of Maspin and Bax was associated with a significant (P-value = 0.015) reduction in tumor mass, while the sole presence of Maspin was not associated with any significant reduction (P-value = 0.250). This significance of the relation between concomitant Maspin and Bax expression with tumor mass reduction could be explained by enhanced tumor cell apoptosis as shown by the occurrence of caspase-3 activation (see Figure 2A).

Similarly (see Figure 5B), the Maspin HSCORE was significantly related to the depth of tumor invasion (P-value = 0.043). The mean values of Maspin HSCORE for each pT-stage were respectively: pT1 = 129.54 ± 25; pT2 = 68 ± 25; pT3 = 48 ± 16.

It is widely accepted that necrosis is frequently associated with the tumor mass. However, and in contrast to the relation of Maspin with tumor mass, we did not find any significant relation to the extent of the necrosis (*P-value *= 0.550). As expected, in our cohort of patients the tumor mass was significant related to the vascular invasion (*P-value *= 0.0018) (Figure 5C). We examined whether Maspin over- expression affected vascular invasion in IHCCA. Our results indicated an absence or presence of vascular invasion when Maspin was above or below the cutpoint, respectively (*P-value *= 0.027) (Figure 5D). The Maspin HSCOREs were 98.3 ± 16 and 39 ± 18 in the absence or in the presence of vascular invasion, respectively. Moreover, the Maspin HSCORE was not related to dysplasia of the intrahepatic bile ducts close to neoplastic lesions, nor with the extent of lymphoid cell infiltration (not shown).

We also wished to verify the potential role of Maspin as an angiogenic inhibitor [30] by evaluating the relation between Maspin expression and microvessel density. The CD34-positive vessel count ranged from 7.5 to 54.4 vessels·mm with a mean value of 34.8 ± 14.1 (median 38). The standardized skewness and kurtosis values (-0.893 and -0.888, respectively) lay within the range expected for data from a normal distribution.

The patients with Maspin HSCORE above the cutpoint had a mean value of CD34- positive vessels of 36.5 ± 14.2 (median 41 vessels) and those below cutpoint had a mean of 32.4 ± 14.3 (median 34 vessels) (*P-value *= 0.5170) indicating a lack of statistical significance.

We finally evaluated the role of Maspin as a prognostic factor [43,44]. The patients who underwent surgery during the last year of enrolment and still alive were excluded from the survival analysis. This choice was supported by the fact that the follow-up period of these patients was shorter than the median length of IHCCA overall survival (18–30 months) [2,45]. The patients with a Maspin HSCORE below the cutpoint had a shorter overall survival (14 ± 10 months) than did patients with Maspin HSCORE above the cutpoint (27 ± 18 months), whereas the log-rank test showed no significant difference between the two groups of patients (*P-value *= 0.162). Moreover, in the evaluation of the relation between Maspin and/or Bax expression and survival there were no statistically significant differences between the three groups (Logrank test *P-value *= 0.483): 37.3 ± 5.7 (Maspin^+^/Bax^+^), 27.2 ± 7.5 (Maspin^+^/Bax^-^), 21.7 ± 3.9 (Maspin^-^/Bax^-^) months, respectively. It should be noted, however that the tumors with an absence of both Maspin and Bax had the shortest mean overall survival.

In our population, the following factors, when analyzed with a univariate binary logistic regression, significantly predicted the risk of death: Stage (P-value = 0.035) and Maspin HSCORE (P-value = 0.025). The multivariable logistic regression (Table 2) including the above-mentioned factors and those parameters with a P-value lower than 0.2 (tumor greatest dimension, Glisson's capsule invasion, invasion of resected margins) indicated that only Maspin HSCORE, invasion of resected margins and stage were independent prognostic factors in IHCCA.

## Discussion

There is good experimental evidence that Maspin abolishes tumor invasion [46], induces tumor re-differentiation [47,48] and also enhances sensitivity of tumor cells to apoptosis by inducing the apoptogenic Bax protein [19,34,36,48]. Furthermore, suppression of Maspin expression by siRNA desensitizes tumor cells to apoptosis [35].

It has been reported that the apoptosis-sensitizing effect of Maspin resulted predominantly from increased Bax expression causing mitochondrial release of apoptogenic factors such as cytochrome C and Smac/DIABLO [19].

We show here that Bax was expressed only when Maspin reached a threshold value (cutpoint). We concluded that Bax expression consequent to a Maspin level above the cutpoint, shifted the relative concentration of anti- *versus *pro-apoptotic members of the Bcl-2 family, thus acting as a positive modulator of the death program. In our IHCCA specimens, however, the concomitant occurrence of Maspin and Bax did not correlate with a better prognosis. In a recent paper [49], the over-expression of Bax in lung cancer specimens suggested a pro-apoptotic cellular tendency that did not correlate with a better prognosis. In contrast, it has been shown [50] in metastatic breast adenocarcinoma, that the expression of Bax protein is associated with increased overall survival and better response to chemotherapy. However, a negative prognostic role of Bax expression has been described in non-small-cell lung cancer [51]. Despite these contradictory results on the role of Bax as a prognostic factor, its induction in Maspin-over-expressing specimens seems to represent an important mechanism by which Maspin delays tumor progression and invasion.

The results presented here show for the first time that the associated expression of Maspin and Bax, detected by immunohistochemistry, significantly correlated with a less aggressive phenotype. Thus, Maspin expression above the cutpoint and the consequent Bax induction can help to explain observations of opposite relations with neoplastic mass, depth of tumor invasion, and vascular invasion. However, it should be noted that Maspin expression was not related with the dysplasia of intrahepatic bile ducts close to the neoplastic lesion, lymphoid cell infiltration, and the extent of necrosis of tumor mass.

We did not find any significant relation between microvessel density and Maspin expression, although somewhat more CD34-positive vessels were observed in those patients with Maspin above the cutpoint. This lack of relation is in contrast with studies showing that Maspin inhibits angiogenesis [52] in vitro and in vivo in several types of tumor. Due to its antiangiogenic effect Maspin has been described as a tumor-suppressor protein. However, our results and a recent report [23] on the expression of Maspin in non-muscle invasive urothelial bladder carcinoma suggest that in IHCCA and some other tumors Maspin may exhibit its tumor-suppressor role by a mechanism unrelated with angiogenesis. Interestingly, it has been shown that tumor neo-vessels become leaky after Maspin treatment [31], whereas normal mature vessels are not affected by Maspin treatment. In addition, it should be noted that our CD34-antibody, a pan-endothelial marker, used to highlight vasculature reveals mainly pre-existing mature vessels and not tumor-induced neovessels.

The caspase-3 activation was found related to the Bax expression in the majority of IHCCA specimens. Interestingly, in all tumor areas with activated caspase-3, a concomitant overexpression of Maspin and Bax was always found. A subset of IHCCA had Bax overexpression without a concomitant activated caspase-3 in tumor cells. A similar finding has been reported in a tumorigenic epithelial cell line [53]. An arrest of the intrinsic apoptotic pathway could occur by different mechanisms such as the impediment of the Bax pro-apoptotic conformational change [36], overexpression of Bax-inhibitor [54], or the prevention of the caspase cascade attributed to overexpression of heat shock proteins (mainly HSP27) [55].

The univariate logistic regression analysis has shown that the factors significantly involved in the risk of death in IHCCA are: staging and Maspin HSCORE. As shown in Table 2, the multivariate logistic analysis indicated that stage, Maspin HSCORE and resected margins' invasion are independent factors for the risk of death. Univariate and multivariate logistic analyses suggest a clear-cut relation between the increased Maspin HSCORE and probability to survive. Our results suggest that a routine assessment of Maspin expression by immunostaining in IHCCA might help detect those patients with a higher risk of death. They are also consistent with the hypothesis, recently described for other types of tumor [43,44,56,57], that reduced/absent Maspin expression may predispose to an increased risk of death. The non-parametric estimates of survival for Maspin HSCORE cutpoint indicate that the mean survival for those patients with HSCORE above the cutpoint was related with longer overall survival although the logrank test showed no significant difference in survival between the patients.

The prognostic relevance of Maspin expression is a matter of debate. For instance, some authors have described Maspin expression as a prognostic marker for several tumors [25,34,44,56-60]. In contrast, other authors feel that Maspin expression [23,61] or overexpression [62] is not a promising prognostic marker.

We also examined Maspin expression on the available liver needle biopsies. Our results (not shown) suggest that immunohistochemical negativity for Maspin clearly indicates a neoplasm with a clinically aggressive course. On the other hand, the immunopositivity is less informative since we cannot exclude the presence of Maspin- negative areas with a lesser degree of differentiation and with aggressive potential.

Our study has the limitation of a low number of patients that is too small to draw valid conclusions or conduct further statistical analysis. The probability of correctly rejecting the null hypothesis (Power of test [63]) did not reach the required amount of power. However, our cohort represents a large single institution series given the rarity of this tumor.

A multicentric and randomized trial will be required to validate our results performed in this explorative retrospective study. If confirmed in a large independent trial, the expression of Maspin should help to discriminate IHCCA patients with a better prognosis. Moreover, pre-operative information retrieved from liver needle biopsies could provide a basis for enroling those patients with a likely unfavorable course for intensive pre-operative management. Finally, it should help devise novel strategies for inducing Maspin expression in IHCCA tumor cells. This procedure could restore tumor cell sensitivity towards a broad range of apoptosis-inducing therapeutic interventions designed to delay tumor progression.

## Conclusion

Maspin expression is negatively associated with IHCCA progression when Bax is expressed. The combined expression of Maspin and Bax may influence the susceptibility of tumor cholangiocytes to apoptosis (as observed by increased caspase-3 expression) and therefore may be involved in delaying IHCCA progression. Procedures designed to induce Maspin expression in IHCCA could restore tumor cell sensitivity towards a broad range of apoptosis-inducing therapeutic interventions and delay tumor progression.

## Competing interests

All authors declare that there are no financial or non-financial competing interests (political, personal, religious, ideological, academic, intellectual, commercial or any other) in relation to this manuscript.

## Authors' contributions

AAR conceived of the study, carried out the design and the immunohistochemical studies, performed the statistical analysis, and wrote the manuscript; AFB conceived of the study, and helped in its design, coordination and critical review of the manuscript; PC supplied the samples, carried out the pathological staging, helped the experimental design and reviewed the manuscript; SD participated in the analysis of results and drafted the manuscript. PS supplied clinical data and reviewed the manuscript. All authors have read and approved the final manuscript.

## Pre-publication history

The pre-publication history for this paper can be accessed here:



## Figures and Tables

**Table 1:** Clinical-pathological features of patients affected by CCA who underwent surgery

*Characteristic*	Patients with Maspin HSCORE above cutpoint (N = 13)	Patients with Maspin HSCORE below cutpoint (N = 9)	P-value
*Age at surgery*	69.3 + 7	67.3 + 10.3	
*Gender*			0.647
Male	5	2	
Female	8	7	
*Tumor greatest dimension*			**0.011**
<4 cm	7	1	
4–6 cm	5	2	
>6 cm	1	6	
*Histologic grade*			0.658
Well/Moderate	9	7	
Poor	4	2	
*Percentage of tumor necrosis*^1#^			0.550
<50	2	3	
51–75	5	2	
>75	6	4	
*Peritumoral border liver*^1#^			0.103
Infiltrated	8	2	
Pushed	5	6	
*Lymphoid infiltration*^1^			0.342
Absent	7	3	
Present	6	6	
*Stage*			0.148
I	4	0	
II	2	1	
III	7	8	

^1^Estimated by pathologist (PC); ^#^data on necrosis and peritumoral liver border status were not available for one patient.

**Table 2:** Multivariate logistic regression analysis.

		***Multivariate Logistic Regression***		
		
***Prognostic factors***	***Adverse Factor***	χ^2^	Df	**P-value**
Tumor greatest dimension	-	0.1335	1	0.7149
Maspin HSCORE	-	3.8940	1	**0.0485**
Resected margins'invasion	**Yes**	4.7674	1	**0.0290**
Glisson's capsule invasion	**Yes **	2.0289	1	0.1543
Stage	**III**	6.1012	2	**0.0473**

Relation between several prognostic factors and risk of death.
